# Gut metabolites identified in cerebrospinal fluid of genetic interferonopathy support gut–brain endothelial dysfunction

**DOI:** 10.1002/cti2.70074

**Published:** 2026-02-19

**Authors:** Russell C Dale, Madysen Elbourne, Markus J Hofer, Shrujna Patel, Shekeeb Mohammad, Velda X Han, Josephine Yu, Shanlin Fu, Arlene D'Silva, Michelle A Farrar, Sushil Bandodkar, Jingya J Yan

**Affiliations:** ^1^ Faculty of Medicine and Health, Clinical School, The Children's Hospital at Westmead University of Sydney Sydney NSW Australia; ^2^ Kids Neuroscience Centre, Faculty of Medicine and Health, The Children's Hospital at Westmead University of Sydney Sydney NSW Australia; ^3^ Faculty of Science, School of Mathematical and Physical Sciences University of Technology Sydney Ultimo NSW Australia; ^4^ Faculty of Science, School of Life and Environmental Sciences, Charles Perkins Centre The University of Sydney Sydney NSW Australia; ^5^ Khoo Teck Puat‐National University Children's Medical Institute National University Health System Singapore Singapore; ^6^ Department of Paediatrics, Yong Loo Lin School of Medicine National University of Singapore Singapore Singapore; ^7^ George Institute University of New South Wales Sydney NSW Australia; ^8^ Department of Neurology The Sydney Children's Hospitals Network Sydney NSW Australia; ^9^ Discipline of Paediatrics and Child Health, School of Clinical Medicine UNSW Medicine and Health, University of New South Wales Sydney NSW Australia; ^10^ Department of Biochemistry The Children's Hospital at Westmead Westmead NSW Australia

**Keywords:** autoinflammatory diseases, cerebrospinal fluid, gut–brain axis, inflammation, metabolomics, serum

## Abstract

**Objective:**

Aicardi–Goutières syndrome (AGS) is a rare genetic interferonopathy because of aberrant DNA or RNA metabolism with secondary host anti‐viral (interferon) activation. This metabolomics study aimed to improve the biological understanding of AGS and explore potential biomarkers.

**Methods:**

We performed untargeted cerebrospinal fluid (CSF) metabolomics using a UPLC‐Q‐Exactive‐HFx Mass Spectrometry of 10 genetically confirmed AGS patients (8 males, mean 4.8 years, range 0.2–16.5) and age‐sex matched controls. Metabolites were then quantified and validated using UHPLC‐QqQ‐MS/MS in CSF and serum.

**Results:**

We identified expected elevated inflammatory metabolites (neopterin and kynurenine) and unexpected elevated gut microbe metabolites in CSF samples: Indole, p‐Cresol, γ‐Butyrobetaine and N‐Butyryl‐L‐homoserine lactone (all *P*_FDR_ < 0.05). Using a targeted assay, we confirmed elevation of these metabolites in CSF, and also in the serum of patients with AGS (all *P* < 0.01).

**Conclusion:**

Our findings suggest gut microbe metabolite leakage traversing the gut–blood–brain barrier in AGS, potentially because of endothelial dysfunction.

## Introduction

Aicardi–Goutières syndrome (AGS) is a rare, monogenic neurodegenerative disorder affecting approximately 1–5 per 10 000 live births.^1^ Initially described as a ‘congenital infection‐like syndrome’, subsequent discovered causative genes were found to be enzymes involved in DNA and RNA breakdown, or proteins involved in RNA sensing in the cell. Loss‐ or gain‐of‐function mutations in these genes trigger an autoinflammatory anti‐viral state with activation of interferon signalling.^2^ AGS is therefore considered a genetic interferonopathy, and to date, most attention has been on the neurological phenotype, but other organ involvement is well‐described, particularly skin inflammation, and cardiopulmonary involvement.^3^ Although gene and RNA therapies hold future promise, current therapeutics remain limited.^4^, ^5^, ^6^ While immune therapies such as JAK inhibitors can reduce the interferon signature, they have an incomplete therapeutic effect on the neurological phenotype.^7^, ^8^, ^9^

We conducted untargeted followed by targeted cerebrospinal fluid (CSF) metabolomics, with subsequent analysis of serum to discover biomarkers in children with AGS, which will help our understanding of disease biology and future therapeutics.

## Results

### Untargeted metabolomics

**Figure 1:**
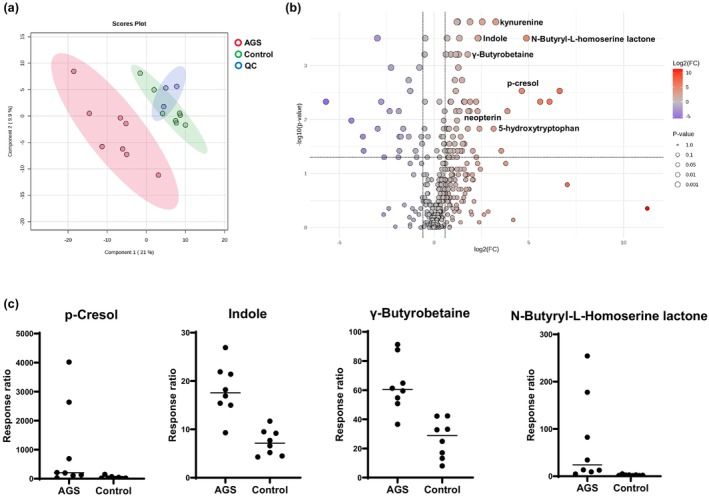
Summary of statistical analyses of CSF untargeted metabolomics case–control study: **(a)** partial least squares discriminant analysis score plots of patients with AGS (red dots), control patients (green dots) and quality control samples (blue dots) showing separation and clustering of the respective groups; **(b)** Volcano plot for AGS and a visual representation of significance and fold change distribution. Upregulated metabolites included the expected pro‐inflammatory metabolites (kynurenine and neopterin) and elevated gut microbial metabolites **(c)** elevation of indole, p‐cresol, γ‐butyrobetaine and N‐butyryl‐L‐homoserine lactone in the AGS group compared to controls, (*P* = 0.01, *P* = 0.04, *P* = 0.01 and *P* = 0.01 respectively, ANOVA and Fisher's LSD FDR‐adjusted). The response ratio is the ratio of *peak area of metabolite: internal standard peak area*.

The PLS‐DA data in our case (*n* = 8) vs. control (*n* = 8) study presented a tight cluster, which indicated good reproducibility and ensured the robustness of the metabolomics analysis method (Figure 1a). Metabolic differences were identified between AGS and controls for 102 CSF metabolites (76 upregulated and 26 downregulated) (Figure 1b). ANOVA and Fisher's LSD *post hoc* analysis revealed 47 metabolites that were significantly involved in driving the discrimination between patients with AGS and controls. Following FDR correction (*P*_FDR_ < 0.05), 19 metabolites were significantly different: 14 metabolites were elevated in the CSF of patients with AGS, and five metabolites were decreased (Table 1). The metabolites were involved in diverse metabolic functions including microbial activity, inflammation, stress responses and neuroprotection.

**Table 1:** Untargeted metabolomics summary of ANOVA and Fisher's least significant difference *post hoc* analysis at a *P*‐value cut‐off of 0.01, fold change and false discovery rate at 5% with *P*_FDR_ adjusted values < 0.05 as statistically significant for children with AGS (*n* = 8) compared to controls (*n* = 8)

Metabolite	Change	Fold change	*P*_FDR_	Metabolite function
**γ‐Butyrobetaine**	**↑**	**2.37**	**0.01**	Gut microbial activity
3,4‐Dimethoxyphenethylamine	**↑**	2.0	0.02	Analogue of dopamine
4‐Hydroxyproline	**↑**	0.35	0.03	Immune modulator
5‐Hydroxytryptophan	**↑**	8.82	0.01	Anti‐inflammatory
Adrenaline	**↑**	2.43	0.02	Inflammation and stress
Asparagine	**↑**	1.60	0.01	Anti‐inflammatory
Carnitine	**↑**	4.65	0.03	Fatty acid metabolism
Deoxyuridine	**↑**	2.35	0.04	DNA metabolism
**Indole**	**↑**	**2.47**	**0.01**	Gut microbial activity
Kynurenine	**↑**	4.68	0.009	Inflammation
**N‐Butyryl‐L‐homoserine lactone**	**↑**	**29.3**	**0.01**	Gut microbial activity
Neopterin	**↑**	2.53	0.01	Inflammation
**p‐Cresol**	**↑**	**24.7**	**0.04**	Gut microbial activity
Serine	**↑**	1.71	0.03	Inflammation
γ‐Glutamylleucine	**↓**	0.27	0.05	Anti‐inflammatory
3‐Methylhistidine	**↓**	0.16	0.05	Protein breakdown
Propanoylagmatine	**↓**	0.51	0.01	Agmatine metabolism
Quisqualic acid	**↓**	0.39	0.02	Excitotoxin
Succinyladenosine	**↓**	0.08	0.04	Purine metabolism

**↑** represents elevation and **↓** represents decreased levels. Gut microbial metabolites in bold.

We identified expected elevation of neuroinflammatory metabolites (kynurenine and neopterin) and anti‐inflammatory metabolites (5‐hydroxytryptophan). Unexpected findings were significantly elevated indole, p‐cresol, γ‐butyrobetaine and N‐butyryl‐L‐homoserine lactone, which are metabolites produced by gut microbes (Figure 1c).

### Targeted validation of gut microbe metabolites in CSF and serum

**Figure 2:**
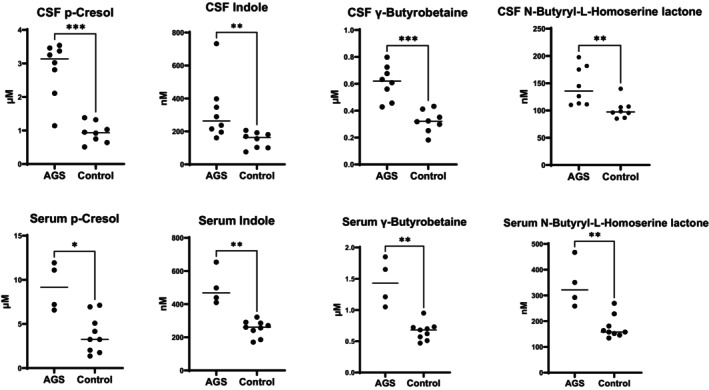
Confirmation of elevated gut microbial metabolomes with targeted quantitative CSF and serum indole, p‐cresol, γ‐butyrobetaine and N‐butyryl‐L‐homoserine lactone. The gut microbial metabolites are elevated in CSF and serum *P < 0.01, **P < 0.005, ***P < 0.0005.

The diversity of chemical and physical properties of the metabolites in Table 1 limited the feasibility of targeted validation in a single assay. Given the unexpected elevation of gut microbial metabolites, we focussed on validating these findings with targeted metabolomics. Using purchased reference standards, we confirmed statistically significant elevations in the AGS group compared to controls of CSF indole (*P* = 0.003), p‐cresol (*P* = 0.0006), γ‐butyrobetaine (*P* = 0.0003) and N‐butyryl‐L‐homoserine lactone (*P* = 0.002) (Mann–Whitney *U* test) (Figure 2). There was no statistical correlation between these CSF microbial metabolites and CSF neopterin, nor any evident gene association driving the findings.

Given the hypothesis that gut microbe metabolites must travel via the blood stream, we tested available serum in AGS patients (*n* = 4) compared to controls (*n* = 9), which confirmed statistically significant elevations of serum indole (*P* = 0.003), p‐cresol (*P* = 0.01), γ‐butyrobetaine (*P* = 0.003) and N‐butyryl‐L‐homoserine lactone (*P* = 0.006) (Mann–Whitney *U* test) (Figure 2).

## Discussion

This study showed the utility of untargeted metabolomics as a discovery‐driven approach to discover metabolite signatures in complex and rare neurological disorders such as AGS. Our study represents the first comprehensive CSF metabolomic analysis in AGS, and we identified expected pro‐inflammatory metabolites but also unexpected gut microbial metabolites, elevated Indole, p‐cresol, γ‐butyrobetaine and N‐butyryl‐L‐homoserine lactone, which were validated using targeted metabolomics.

The classic biomarkers of AGS include increased interferon‐alpha (IFN‐α) activity in CSF,^10^ elevated CSF neopterin,^11^ interferon stimulated genes RNA panel in blood^12^ and confirmatory gene testing.^13^ Previous ‘omics’ research in human AGS,^14^, ^15^, ^16^, ^17^ primarily investigating the transcriptome and proteome of blood and CSF, has identified mitochondrial dysfunction, upregulation of toll‐like receptor pathways, changes in synaptic protein expressions and micronuclei formation, and disrupted nucleic acid metabolism.

The gut microbiome is one of the most chemically diverse ecosystems in the human body, playing crucial roles in metabolic regulation, endocrine signalling and immune modulation.^18^ Precursors of uremic toxins, indole and p‐cresol are gut‐derived microbial metabolites formed by the fermentation of the amino acids tryptophan and tyrosine, respectively, by proteolytic gut bacteria.^19^ γ‐Butyrobetaine is an intermediate in the gut microbial metabolism of L‐carnitine to trimethylamine‐*N*‐oxide. The elevated formation of γ‐butyrobetaine has been implicated in atherosclerosis^20^ and peripheral artery disease.^21^ N‐Butyryl‐L‐homoserine lactone is a bacterial signalling molecule involved in quorum sensing within the gut microbiome, coordinating gene expression and physiology of bacterial populations. N‐Butyryl‐L‐homoserine lactone has been shown to interact with inflammation pathways in inflammatory bowel disease^22^, ^23^ and is positively associated with the production of butyrate.^24^

Microbial metabolite levels can vary between individuals because of differences in gut microbiota composition influenced by diet, genetics and the environment;^25^ however, under normal physiological conditions, these metabolites are typically present in the CSF only at trace levels.^26^ Simultaneous analysis of indole, p‐cresol, γ‐butyrobetaine and N‐butyryl‐L‐homoserine lactone in serum revealed statistically significant elevations consistent with those observed in CSF. This cross‐compartmental profiling revealed elevated gut‐derived metabolites in both central and peripheral systems, which supports the concept of altered blood–brain barrier permeability and leakage from gut into blood and blood into brain compartments.^27^

DNA and RNA metabolism is a key feature of all nuclei containing cells, and although the brain appears specifically vulnerable in AGS, broad cellular dysfunction can be expected in AGS. Mutations in DNA and RNA metabolism cause persistent interferon signalling and secondary activation of brain‐derived endothelial cells. The endothelial cells show increased interferon stimulatory genes and release proinflammatory cytokines which promote vascular inflammation and injury,^28^, ^29^ contributing to microangiopathy, microinfarctions in AGS that is seen in histopathology, and clinical cerebral small vessel disease plus white matter injury.^30^ The endothelium is a key barrier between the gut and blood, and blood and brain, and these gut metabolites may transpire to be useful biomarkers in the assessment of endothelial integrity.

Limitations of the study are the limited sample size, the lack of a validation cohort and other complementary studies to examine endothelial integrity in AGS patients compared to controls. An inherent challenge of CSF biomarker discovery is the challenge of obtaining ‘healthy’ CSF, and the controls used in this study were being investigated for neurological disease, although there was no evidence of neuroinflammation. Fasting and feeding adequacy could also influence gut microbial values in serum. Although all samples were taken after a minimum of 4 h of fasting, it is known that children with AGS can experience feeding difficulties, which could potentially have confounded the findings.

A further improvement to our study would have included examination of the gut microbiome to see whether the AGS patients have a different resident microbiome, which could theoretically influence our findings. We recognise that the crosstalk between the immune system and gut microbiota is complex and exhibits great individual variability presenting challenges in establishing cause and effect relationships. Future studies can examine these metabolites in other neurological conditions thought to have ‘leaky gut’ such as autism and explore whether the gut microbial metabolites could have a contributory pro‐inflammatory effect in the blood or brain.

## Methods

### Study design and participants

Ten patients with genetically confirmed AGS, who were on no immune therapies, were recruited (2 females, 8 males, mean 4.8 years, range 0.2–16.5 years) (Table 2): Eight had adequate CSF available for untargeted followed by targeted metabolomics, and serum was available in four. The patients were not acutely unwell at the time of sampling.

**Table 2:** Clinical data of AGS patients *n* = 8 including demographics, sample (CSF, serum used in studies), genetic mutation, clinical, radiological and CSF findings

Age at CSF/sex	CSF	Serum	Genetic mutation		Symptom onset	Neurological symptoms	MRI	CSF pleocytosis	CSF neopterin (nmol/L)
2 years 6 months/F	+		TREX1 c.341G>A (p.Arg114His)	Homozygous	3 weeks	ID, regression, spasticity, dystonia, microcephaly, IUGR, cardiomyopathy, visual impairment, chilblains	Periventricular, deep white matter calcifications	Nil	2024
4 years 8 months/M	+		RNASEH2B c.529G>A (p.Ala177Thr)	Homozygous	13 months	ID, regression, spasticity, dystonia	T2 hyperintensities in frontal and parietal periventricular white matter	Nil	242
3 years 3 months/M	+	+	ADAR1 c.3019G>A (p.Gly1007Arg)	Heterozygous	11 months	Regression, spasticity, dystonia	Hyperintensity and mild atrophy of putamen and caudate nucleus	Nil	81
6 years/F	+		ADAR1 c.1078C>T (p.Arg360Ter) and c.577C>G (p.Pro193Ala)	Heterozygous	6 months	ID, regression, spasticity, dystonia, microcephaly	Progressive leukodystrophy	Nil	145
14 years/M	+		ADAR1 c.3019G>A (p.Gly1007Arg)	Heterozygous	2 years	Regression, dystonia	Mild atrophy of bilateral posterior putamina	Nil	68
2 years/M	+	+	RNASEH2B c.529G>A (p.Ala177Thr)*	Compound heterozygote	3 weeks	ID, spasticity, dystonia, microcephaly, recurrent fevers	High signal intensity in subcortical white matter, cerebral atrophy	81 mononuclear cells	1410
3 years/M	+		SAMHD1 c.433C>T (p.R145X) and c.490C>T (p.R164X)	Compound heterozygote	3 months	ID, regression, spasticity, dystonia, microcephaly	Leukodystrophy of white matter, cerebral atrophy	Nil	1102
0.5 year/M	+		TREX1 c.389A>C (p.Asp130Ala)	Homozygous	Neonatal	ID, regression, spasticity, dystonia, microcephaly	Leukodystrophy of white matter, cerebral atrophy	Nil	1792
7 years/M		+	IFIH1 c.2335C>T (p.Arg779Cys)	*De novo* heterozygote	8 months	ID, regression, spasticity, dystonia, microcephaly	Normal	Nil	98
5 years/M		+	IFIH1 c.1178A>C (p.Asp393Ala)	*De novo* heterozygote	6 weeks	ID, regression, spasticity, dystonia, microcephaly	Non‐specific T2 hyper intensity in parietal white matter	Nil	306

* applies to genetic nomenclaure.

Nine age‐, sex‐matched controls were recruited (2 females, 7 males, mean 4.35 years, range 0.5–10.1 years) (CSF *n* = 8, serum *n* = 9). The controls had idiopathic intracranial hypertension (*n* = 4), delayed development (*n* = 3), and suspected epilepsy (*n* = 2). All controls had normal routine CSF investigation with normal neopterin, and none had identified neuroinflammation.

CSF and serum samples in patients and controls were taken under general anaesthetic after a minimum of 4 h of fasting. All CSF samples were collected using an aseptic technique and frozen within 1 h of sampling and stored at −80°C. The CSF tube analysed for this study was not used for routine testing and was not previously thawed before the metabolomic studies. All serum samples were stored at −40°C until use.

### Chemical and reagents

For untargeted metabolomics, an internal standard mixture of d3‐tryptophan and d4‐kynurenine (Toronto Research Chemicals, Toronto, Canada) was used. For the targeted metabolomics, indole, p‐cresol, γ‐butyrobetaine and N‐butyryl‐L‐homoserine lactone were purchased as powders from Sapphire Bioscience (Sydney, Australia). A mixed internal standard consisting of d_7_‐indole, d_7_‐p‐cresol and d_5_‐N‐butyryl‐L‐homoserine lactone was purchased from Sapphire Bioscience (Sydney, Australia). HPLC grade acetonitrile, methanol and ammonium formate were obtained from Sigma‐Aldrich (Sydney, Australia). Formic acid was purchased from Fisher Chemical (Fair Lawn, New Jersey). The catalogue numbers of the chemicals and reagents are provided in Supplementary table 1.

### Untargeted metabolomics

The sample preparation and metabolomic profiling were performed in accordance with the previously reported method of Yan *et al.* ^31^ Briefly, 100 μL of human CSF was deproteinised with methanol, then vortexed, sonicated and centrifuged. The supernatant was dried under nitrogen and reconstituted prior to analysis on a Thermo Scientific Vanquish system coupled to a Q Exactive HF‐X Hybrid Quadrupole–Orbitrap mass spectrometer (Thermo Fisher Scientific, San Jose, CA, USA). Metabolite separation was performed with an Acquity UPLC HSS T3 Column (2.1 mm × 150 mm 1.7 μm particle size), at a flow rate of 0.30 mL min^−1^ and a 25‐min gradient program. The mass spectrometer was operated in positive electrospray mode with all‐ion fragmentation, at a high mass resolution of 120 000 and a scan range from m/z 60–700. Three pooled quality controls were analysed.

Peak detection and alignment, retention time correction and metabolite identification were performed on the raw data using Compound Discoverer 3.4 (Thermo Fisher Scientific). The pre‐processed raw data were normalised against internal standards. Features detected in fewer than 75% of samples were eliminated to reduce false positives and negatives. Retention time alignment was set to a 0.2‐min shift window with a mass accuracy threshold of 5 ppm, and features were extracted using a minimum absolute intensity threshold of 5000 counts across a m/z window of 70–500. Metabolites were annotated using the Human Metabolome Database, KEGG, an in‐house built database and mzCloud using a 5 ppm mass tolerance.

Multivariate and univariate analyses were undertaken using MetaboAnalyst 6.0. The multivariate analyses included partial least squares discriminant analysis (PLS‐DA), a volcano plot and fold change analysis. Statistically significant metabolites discriminating AGS from the control group were determined by ANOVA and Fisher's least significant difference (LSD) *post hoc* testing, applying a significance threshold of *P* < 0.01 and false discovery rate at 5% with *P*_FDR_ values < 0.05.

### Targeted quantification of gut metabolites in CSF and serum

CSF levels of indole, p‐cresol, γ‐butyrobetaine and N‐butyryl‐L‐homoserine lactone were quantified in all patients with AGS (*n* = 8) and controls (*n* = 8) from the untargeted case–control study. These metabolites were also quantified in serum of AGS patients (*n* = 4) and controls (*n* = 9). Indole, p‐cresol, γ‐butyrobetaine and N‐butyryl‐L‐homoserine lactone were extracted from human CSF and serum samples using the sample preparation protocol. 100 μL of human sample was deproteinised with 400 μL of methanol: acetonitrile (1:1 v/v) in Nanosep 0.2 μM centrifugal devices. The mixed IS solution was added to the precipitation reagent resulting in a final concentration of 200 ng mL^−1^. The CSF samples were vortexed and precipitated in ice at 5°C. It is followed by centrifugation for 10 min at a temperature of 5°C and velocity of 6000 g. The supernatant was dried under nitrogen and reconstituted for analysis using a Thermo Scientific Vanquish system coupled to a TSQ Altis triple quadrupole mass spectrometer (Thermo Fisher Scientific, San Jose, CA, USA). Metabolites were separated chromatographically using the Acquity UPLC HSS T3 Column (2.1 mm × 150 mm 1.7 μm particle size) at a flow rate of 0.30 mL min^−1^ using a 12‐min gradient program. The mobile phases consisted of 0.02% formic acid and 10 mM ammonium formate (A) and 0.02% formic acid and 10 mM ammonium formate in acetonitrile (B). The gradient program was as follows: 0–1 min (5% B), 1–5 min (5–95% B), 5–9 min (95% B), 9–9.5 min (95–5% B) and 9.5–12 min (5% B). Metabolites were detected by the mass spectrometer in MRM mode with positive electrospray ionisation. Peaks were integrated using Xcalibur and normalised to their corresponding internal standard.

### Statistics

Statistical analyses were conducted using GraphPad Prism 8 and SPSS version 26. As the data were not normally distributed, nonparametric statistics (Mann–Whitney *U* test) were applied.

### Ethics

The study received ethics approval from the Sydney Children's Hospitals Network Human Research Ethics Committee (2022/ETH00574 and 2019/ETH06182). Families provided written informed consent for the use of residual CSF remaining after routine diagnostic testing.

## Author contributions

RCD, SB, JJY, SP, SF and AD conceptualised and designed the experiments. RCD, VXH, SM, MAF recruited and phenotyped study patients. JJY, ME, JY performed the experiments and data acquisition. JJY, RCD analysed and interpreted the data. JJY and RCD performed validation. JJY, RCD, MJH wrote the original manuscript. All authors critically revised and edited the manuscript. All authors approved the final version of the article.

## Conflict of interest

The authors declare no conflict of interest.

## Supporting information


Supplementary table 1

## Data Availability

Deidentified datasets generated and analysed in this study are available from the corresponding author upon reasonable request. Access to the raw data requires submission of a written study proposal, subject to approval by the Kids Neuroscience Centre.

## References

[1] Møller RS , Zhao L , Shoaff JR *et al*. Incidence of Aicardi‐Goutières syndrome and KCNT1‐related epilepsy in Denmark. Mol Genet Metab Rep 2022; 33: 100924. 36262748 10.1016/j.ymgmr.2022.100924 PMC9574483

[2] Crow YJ , Stetson DB . The type I interferonopathies: 10 years on. Nat Rev Immunol 2022; 22: 471–483. 34671122 10.1038/s41577-021-00633-9 PMC8527296

[3] Adang LA , Frank DB , Gilani A *et al*. Aicardi goutières syndrome is associated with pulmonary hypertension. Mol Genet Metab 2018; 125: 351–358. 30219631 10.1016/j.ymgme.2018.09.004 PMC6880931

[4] Viengkhou B , Hong C , Mazur C *et al*. Interferon‐α receptor antisense oligonucleotides reduce neuroinflammation and neuropathology in a mouse model of cerebral interferonopathy. J Clin Invest 2024; 134: e169562. 38357922 10.1172/JCI169562 PMC10869178

[5] Dell'Isola GB , Dini G , Culpepper KL *et al*. Clinical spectrum and currently available treatment of type I interferonopathy Aicardi‐Goutières syndrome. World J Pediatr 2023; 19: 635–643. 36650407 10.1007/s12519-022-00679-2 PMC10258176

[6] Crow YJ , Shetty J , Livingston JH . Treatments in Aicardi‐Goutières syndrome. Dev Med Child Neurol 2020; 62: 42–47. 31175662 10.1111/dmcn.14268

[7] Politano D , Tonduti D , Battini R , Fazzi E , Orcesi S . Exploring emerging JAK inhibitors in the treatment of Aicardi‐Goutières syndrome. Expert Opin Emerg Drugs 2024; 30: 21–39. 10.1080/14728214.2024.2445508 39704072

[8] Jafarpour S , Suddock J , Hawes D , Santoro JD . Neuropathologic impacts of JAK inhibitor treatment in Aicardi‐Goutières syndrome. J Clin Immunol 2024; 44: 68. 38381212 10.1007/s10875-024-01672-2

[9] Vanderver A , Adang L , Gavazzi F *et al*. Janus kinase inhibition in the Aicardi‐Goutières syndrome. N Engl J Med 2020; 383: 986–989. 32877590 10.1056/NEJMc2001362 PMC7495410

[10] Lodi L , Melki I , Bondet V *et al*. Differential expression of interferon‐alpha protein provides clues to tissue specificity across type I interferonopathies. J Clin Immunol 2021; 41: 603–609. 33411153 10.1007/s10875-020-00952-x

[11] Han VX , Mohammad SS , Jones HF , Bandodkar S , Crow YJ , Dale RC . Cerebrospinal fluid neopterin as a biomarker of treatment response to Janus kinase inhibition in Aicardi‐Goutières syndrome. Dev Med Child Neurol 2022; 64: 266–271. 34415581 10.1111/dmcn.15025

[12] Rice GI , Forte GM , Szynkiewicz M *et al*. Assessment of interferon‐related biomarkers in Aicardi‐Goutières syndrome associated with mutations in TREX1, RNASEH2A, RNASEH2B, RNASEH2C, SAMHD1, and ADAR: A case‐control study. Lancet Neurol 2013; 12: 1159–1169. 24183309 10.1016/S1474-4422(13)70258-8 PMC4349523

[13] Crow YJ , Chase DS , Lowenstein Schmidt J *et al*. Characterization of human disease phenotypes associated with mutations in TREX1, RNASEH2A, RNASEH2B, RNASEH2C, SAMHD1, ADAR, and IFIH1. Am J Med Genet A 2015; 167a: 296–312. 25604658 10.1002/ajmg.a.36887 PMC4382202

[14] Dragoni F , Garau J , Sproviero D *et al*. Characterization of mitochondrial alterations in Aicardi‐Goutières patients mutated in RNASEH2A and RNASEH2B genes. Int J Mol Sci 2022; 23: 14482. 36430958 10.3390/ijms232214482 PMC9692803

[15] Ramantani G , Kohlhase J , Hertzberg C *et al*. Expanding the phenotypic spectrum of lupus erythematosus in Aicardi‐Goutières syndrome. Arthritis Rheum 2010; 62: 1469–1477. 20131292 10.1002/art.27367

[16] Cattalini M , Galli J , Andreoli L *et al*. Exploring autoimmunity in a cohort of children with genetically confirmed Aicardi‐Goutières syndrome. J Clin Immunol 2016; 36: 693–699. 27539236 10.1007/s10875-016-0325-y

[17] Giordano AMS , Luciani M , Gatto F *et al*. DNA damage contributes to neurotoxic inflammation in Aicardi‐Goutières syndrome astrocytes. J Exp Med 2022; 219: e20211121. 35262626 10.1084/jem.20211121 PMC8916121

[18] Cryan JF , O'Riordan KJ , Sandhu K , Peterson V , Dinan TG . The gut microbiome in neurological disorders. Lancet Neurol 2020; 19: 179–194. 31753762 10.1016/S1474-4422(19)30356-4

[19] Candeliere F , Simone M , Leonardi A , Rossi M , Amaretti A , Raimondi S . Indole and p‐cresol in feces of healthy subjects: Concentration, kinetics, and correlation with microbiome. Front Mol Med 2022; 2: 959189. 39086966 10.3389/fmmed.2022.959189 PMC11285674

[20] Koeth RA , Levison BS , Culley MK *et al*. γ‐Butyrobetaine is a proatherogenic intermediate in gut microbial metabolism of L‐carnitine to TMAO. Cell Metab 2014; 20: 799–812. 25440057 10.1016/j.cmet.2014.10.006 PMC4255476

[21] Chen ZW , Wu WK , Chiang JY , Cheng NC , Lee JK , Wu MS . Microbiota γ‐Butyrobetaine is associated with increased risk of major adverse limb events in people with lower extremity arterial disease undergoing endovascular therapy. J Am Heart Assoc 2025; 14: e037356. 40820985 10.1161/JAHA.124.037356 PMC12748064

[22] Coquant G , Grill JP , Seksik P . Impact of N‐acyl‐homoserine lactones, quorum sensing molecules, on gut immunity. Front Immunol 2020; 11: 1827. 32983093 10.3389/fimmu.2020.01827 PMC7484616

[23] Grellier N , Suzuki MT , Brot L *et al*. Impact of IBD‐associated dysbiosis on bacterial quorum sensing mediated by acyl‐homoserine lactone in human gut microbiota. Int J Mol Sci 2022; 23: 15404. 36499731 10.3390/ijms232315404 PMC9738069

[24] Chen Y , Chen T , Yin J . Impact of N‐butyryl‐l‐homoserine lactone‐mediated quorum sensing on acidogenic fermentation under saline conditions: Insights into volatile fatty acids production and microbial community. Bioresour Technol 2023; 368: 128354. 36410593 10.1016/j.biortech.2022.128354

[25] Kim CS . Roles of diet‐associated gut microbial metabolites on brain health: Cell‐to‐cell interactions between gut bacteria and the central nervous system. Adv Nutr 2024; 15: 100136. 38436218 10.1016/j.advnut.2023.10.008 PMC10694655

[26] Dando SJ , Mackay‐Sim A , Norton R *et al*. Pathogens penetrating the central nervous system: Infection pathways and the cellular and molecular mechanisms of invasion. Clin Microbiol Rev 2014; 27: 691–726. 25278572 10.1128/CMR.00118-13 PMC4187632

[27] Aburto MR , Cryan JF . Gastrointestinal and brain barriers: Unlocking gates of communication across the microbiota‐gut‐brain axis. Nat Rev Gastroenterol Hepatol 2024; 21: 222–247. 38355758 10.1038/s41575-023-00890-0

[28] Cuadrado E , Michailidou I , van Bodegraven EJ *et al*. Phenotypic variation in Aicardi‐Goutières syndrome explained by cell‐specific IFN‐stimulated gene response and cytokine release. J Immunol 2015; 194: 3623–3633. 25769924 10.4049/jimmunol.1401334

[29] Viengkhou B , Hayashida E , McGlasson S *et al*. The brain microvasculature is a primary mediator of interferon‐α neurotoxicity in human cerebral interferonopathies. Immunity 2024; 57: 1696–1709.e1610. 38878770 10.1016/j.immuni.2024.05.017 PMC11250091

[30] Barth PG . The neuropathology of Aicardi‐Goutières syndrome. Eur J Paediatr Neurol 2002; 6 Suppl A: A27–A31; discussion A37‐29, A77‐86. 12365358 10.1053/ejpn.2002.0570

[31] Yan J , Han VX , Jones HF *et al*. Cerebrospinal fluid metabolomics in autistic regression reveals dysregulation of sphingolipids and decreased β‐hydroxybutyrate. EBioMedicine 2025; 114: 105664. 40138886 10.1016/j.ebiom.2025.105664 PMC11986237

