# Supplementary material for: Gut metabolites identified in cerebrospinal fluid of genetic interferonopathy support gut–brain endothelial dysfunction
Source: Clin Transl Immunology. 2026 Feb 19;15(2):e70074. doi: 10.1002/cti2.70074 (PMC12920058; doi:10.1002/cti2.70074)
Supplement: Supplementary file 1 — Supplementary table 1 [file CTI2-15-e70074-s001.docx]

# Supplemental Table 1: Catalogue number for chemicals and reagents.

| **Chemical/ Reagent** | **Product Number** | **Manufacturer** |
| --- | --- | --- |
| Acetonitrile (HPLC grade) | 34851 | Sigma Aldrich (Sydney, Australia) |
| Methanol (HPLC grade) | 34860 |  |
| Ammonium formate | 70221 |  |
| tert-Butyl methyl ether | 34875 |  |
| d_3_-Tryptophan | T947202 | Toronto Research Chemicals (Toronto, Canada) |
| d_4_-Kynurenine | K661003 |  |
| d_7_-Indole | 1577331 | Sapphire Bioscience (Sydney, Australia) |
| d_7_-p-Cresol | C781902 |  |
| d_5_-N-Butyryl-L-homoserine lactone | 10007899 |  |
| Indole | I577320 |  |
| p-Cresol | C781900 |  |
| N-Butyryl-L-homoserine lactone | 10007898 |  |
| γ-Butyrobetaine | B759500 |  |
| Formic acid | T27563 | Fisher Chemical (Fair Lawn, New Jersey) |
